# Evaluating Viscoelastic Properties of the Wrist Joint During External Perturbations: Influence of Velocity, Grip, and Handedness

**DOI:** 10.3389/fnhum.2021.726841

**Published:** 2021-10-04

**Authors:** Valeria Falzarano, Michael W. R. Holmes, Lorenzo Masia, Pietro Morasso, Jacopo Zenzeri

**Affiliations:** ^1^Department of Informatics, Bioengineering, Robotics, and Systems Engineering, University of Genova, Genova, Italy; ^2^Robotics, Brain, and Cognitive Sciences, Istituto Italiano di Tecnologia, Genova, Italy; ^3^Faculty of Applied Health Sciences, Brock University, St. Catharines, ON, Canada; ^4^Institut für Technische Informatik, Universität Heidelberg, Heidelberg, Germany

**Keywords:** wrist mechanical impedance, viscoelastic properties, wrist stiffness, robotic assessment, grip force, handedness

## Abstract

In this study, we designed a robot-based method to compute a mechanical impedance model that could extract the viscoelastic properties of the wrist joint. Thirteen subjects participated in the experiment, testing both dominant and nondominant hands. Specifically, the robotic device delivered position-controlled disturbances in the flexion-extension degree of freedom of the wrist. The external perturbations were characterized by small amplitudes and fast velocities, causing rotation at the wrist joint. The viscoelastic characteristics of the mechanical impedance of the joint were evaluated from the wrist kinematics and corresponding torques. Since the protocol used position inputs to determine changes in mean wrist torque, a detailed analysis of wrist joint dynamics could be made. The scientific question was whether and how these mechanical features changed with various grip demands and perturbation velocities. Nine experimental conditions were tested for each hand, given by the combination of three velocity perturbations (fast, medium, and slow) and three hand grip conditions [self-selected grip, medium and high grip force, as percentage of the maximum voluntary contraction (MVC)]. Throughout the experiments, electromyographic signals of the extensor carpi radialis (ECR) and the flexor carpi radialis (FCR) were recorded. The novelty of this work included a custom-made soft grip sensor, wrapped around the robotic handle, to accurately quantify the grip force exerted by the subjects during experimentation. Damping parameters were in the range of measurements from prior studies and consistent among the different experimental conditions. Stiffness was independent of both direction and velocity of perturbations and increased with increasing grip demand. Both damping and stiffness were not different between the dominant and nondominant hands. These results are crucial to improving our knowledge of the mechanical characteristics of the wrist, and how grip demands influence these properties. This study is the foundation for future work on how mechanical characteristics of the wrist are affected in pathological conditions.

## Introduction

Upper extremity movements are crucial elements of daily life and our sensorimotor system is capable of coordinating complex movements with apparent ease. However, the underlying computational complexity required to successfully execute upper extremity tasks has challenged motor scientists in the last decades. In particular, wrist movements are functionally critical because the wrist is at the end of a kinematic chain, and thus, control is fundamental for limb accuracy in most tasks. In tasks that require accurate actions, e.g., pointing, reaching, and pushing, humans tend to reduce the source of inaccuracy by choosing appropriate arm postures that stiffen all the degrees of freedom (DoF) of the arm, except the wrist. Charles and Hogan (2010) demonstrated that the trajectory and control of the wrist rotations are influenced by the wrist stiffness. Since wrist movements are dominated by the viscoelastic characteristics of the wrist joint (Charles and Hogan, 2012), knowledge of these characteristics becomes essential to understand how humans perform coordinated wrist rotational movements (Formica et al., 2012; Drake and Charles, 2014). Formica and colleagues estimated passive stiffness of the wrist joint, i.e., resistance to stretching in the absence of muscle activity, confirming low muscle activity and balanced coactivation, supported by the provision of posture stability by the robot. In this study, they demonstrated anisotropy of passive stiffness, suggesting that one purpose of wrist motor control involves compensating for passive stiffness of the wrist by rotating the joint along the direction of least stiffness. As mentioned above, muscle stiffness is not only a load to be compensated for by the motor control, but also a fundamental mechanism of control itself, within the framework of the equilibrium point hypothesis, impedance control, and other types of control (Bizzi et al., 1992; Feldman and Levin, 1995). The aim of our work was to implement a quantitative assessment for the resistance of the wrist joint to external perturbations, often referred to as mechanical impedance (Hogan, 1990) (i.e., the dynamical relationship between wrist kinematics and torque). Taking into account the ability to quantitatively record kinematic data through robotic systems (Colombo et al., 2005; Salehi Dehno et al., 2020), we propose a robotic measurement protocol that delivers sequences of perturbations along the flexion-extension direction of the wrist as in Falzarano et al. (2020) to compute the dynamic wrist joint viscoelastic properties. Dynamic is associated with our protocol for evaluating the different components of the mechanical impedance and refers to the fact that the perturbation is sufficient to exclude modifications of the central neural drive. This contrasts with other protocols that use a very slow perturbation which cannot exclude a neural component.

From previous studies on the evaluation of the mechanical characteristics of the arm (Mussa-Ivaldi et al., 1985; Tsuji et al., 1995; Burdet et al., 2001), stiffness emerged to be strongly related to the grip force of the subject and the subsequent coactivation. Indeed, increases in grip force are largely produced by forearm muscle contraction, which has been demonstrated to impact wrist joint stiffness (Holmes et al., 2015). Additionally, an increase in joint stiffness represents a major source of disability and one of the most common symptoms of neuromuscular disorders (Vattanasilp et al., 2000), such as spasticity and rigidity. Stiffness evaluation is crucial for proper diagnostic assessment, optimal rehabilitation protocols, and for monitoring patients’ progress and therapeutic efficacy. Nevertheless, in the clinical context, spasticity is evaluated using clinical scales based on manual tests of resistance to stretch such as the Modified Ashworth Scale (MAS) and the Modified Tardieu Scale (MTS), which have been shown to be unreliable and influenced by the operator’s capabilities (Ansari et al., 2006; Abolhasani et al., 2012). Thus, a quantitative evaluation of the mechanical impedance at the wrist joint and the characterization of its viscoelastic properties are likely to be very useful in clinics. Thus, in light of extending this experimental protocol to clinical settings, in particular to quantify spasticity in patients affected by neurological disorders, we decided to further investigate the effect of perturbation velocity on the viscoelastic properties of the wrist joint. Specifically, it is well known that spasticity is related to stiffness and is velocity-dependent, as reported by Lance (1980). For all the above-mentioned reasons, we chose to closely monitor how the viscoelastic characteristics of the healthy human wrist is affected by grip demand, perturbation velocity, and handedness.

## Materials and Methods

### Participants and Experimental Setup

**FIGURE 1:**
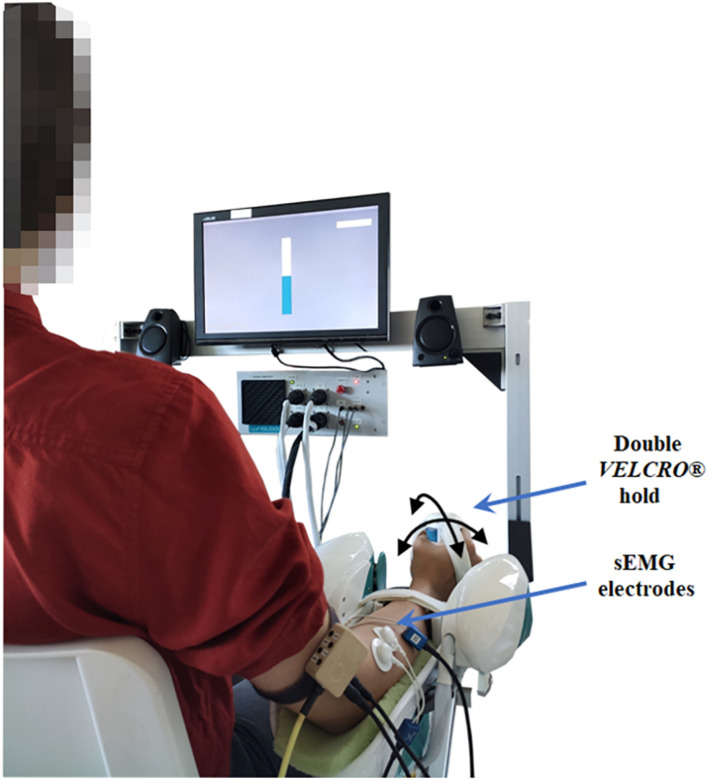
A schematic representation of the participant, bipolar electrodes placement and robot position in the experimental setup. An example of the visual feedback of the grip force is shown through a virtual bar on the screen in front of the participant.

Thirteen volunteers (6 females, 7 males; 27.2 ± 1.9 years of age) were recruited for the experiment. All participants were right-handed and had no known neurological disorders or previous wrist injuries. Each participant signed informed consent before participating in the study. The experimental protocol was performed at the Motor Learning, Assistive, and Rehabilitation Robotics Lab of the Istituto Italiano di Tecnologia and was approved by the local ethical committee of the Liguria Region (n. 222REG2015), following the Declaration of Helsinki principles. During the experiment, participants sat on a chair in front of a computer screen and grasped the handle of the robot. The robotic device used for this study is the WristBot (Masia et al., 2009; Iandolo et al., 2019), a manipulandum that allows wrist movements along three DoF: flexion/extension (FE), radial/ulnar deviation (RUD), and pronation/supination (PS), with a range of motion (RoM) for each DoF comparable to humans (Figure 1). The device, which was recently re-engineered in order to improve the mechanical robustness and the control system, is equipped with four brushless motors, activated by a control unit that allows two modes of operation: position or torque control, in such a way to deliver position or torque perturbations to the subjects, according to specific experimental protocols. Specifically, the control unit was programmed to generate a positional perturbation along the FE DoF, whereas the two other DoFs were position-controlled to maintain firmly the neutral angular value. FE rotations, as well as the residual small movements of the other DoFs, were measured with high-resolution incremental encoders integrated into the WristBot actuators at a 1 kHz sample rate.

The robotic handle was wrapped with a custom-made soft sensor that could measure the grip force exerted by the subjects, while an integrated virtual reality environment provided visual feedback of grip intensity to the user. In particular, the custom-made grip sensor is composed of a polyurethane foam covered with a nickel-copper wire material. When the sensor is compressed, the electrical resistance of the foam decreases. Changes in resistance are converted to an analog signal, expressed in Volts, through an external electric circuit and sent to the electronic board of the robot. The custom-made grip sensor is glued to a cylindrical plastic support that precisely fits the robot handle. A calibration procedure was performed by asking each subject to exert a maximum grip force first on a hand-held hydraulic dynamometer (Baseline 7-Piece Hand Evaluation Kit, Fabrication Enterprises) and then on the sensorized robot handle. Assuming a linear relationship between the custom sensor voltage and the grip force, it was possible to directly relate the grip force measured in Newtons (hydraulic dynamometer) to the change in voltage (recorded via the custom sensor).

During the experiments, surface electromyography (sEMG) was recorded from two forearm muscles of the arm under examination, including extensor carpi radialis (ECR) and flexor carpi radialis (FCR). The corresponding sEMG signals were acquired using bipolar Ag/AgCl electrodes, with a sampling rate of 2048 Hz (OTBioelettronica Quattrocento). Electrode preparation and placement followed the SENIAM protocol (Surface Electromyography for the Non-Invasive Assessment of Muscles) (Hermens et al., 1999). The kinematics, torques, and sEMG signals were synchronized by a trigger signal from the robotic device to the sEMG measurement system to relate the muscle activation to the corresponding movement.

### Experimental Protocol

Before beginning the experimental procedures with each subject, the experimenters carried out five preliminary operations:

(1) Skin cleansing and placement of the bipolar electrodes over the muscle belly of ECR and FCR.
(2) Adaptation of the experimental setup to the subject’s body parameters and explanation of the correct manner to sit, position the arm and grasp the handle. As shown in Figure 1, subjects were positioned with the arm under investigation externally rotated (approximately 20°) and the longitudinal position of the robot was adjusted to produce an elbow extension angle of approximately 120°; additionally, the forearm in a neutral position (midway between pronation and supination) was firmly attached to the robotic manipulandum through a pair of *VELCRO*^®^ straps that prevented forearm movements, avoided slacking motions between hand and handle and isolated the wrist joint. For the placement of the wrist in its neutral position we aligned the third metacarpal approximately parallel to the long axis of the radius (Wu et al., 2005).
(3) Evaluation of the grip force during maximum voluntary contraction (MVC) with two methods: (1) with the hydraulic hand-held dynamometer and (2) with the sensorized robot handle. For each subject, grip sensor data were then normalized as %MVC.
(4) Evaluation of the maximum voluntary excitation (MVE) during maximal isometric wrist flexion and extension contractions. Maximal contractions lasted 2 s, were repeated three times and used in the post-processing for the muscle specific normalization of the sEMG signals (%MVE).
(5) Encouraging each subject to be engaged in a familiarization phase, in order to feel comfortable with the experimental conditions.

After familiarization, the experimental protocol began. For each subject, the experimental setup was arranged to investigate either the right (RH) and left (LH) hands with a random starting order. The preparation phase and the following experimental procedure were the same for both hands but, for convenience, we limit the presentation to one hand only. Specifically, the robot generated a pseudo-random sequence of angular perturbations of small amplitude, compatible with the linearity of the model of the mechanical impedance of the wrist adopted in this study. The angular perturbations were symmetrical with respect to the neutral wrist position along the FE DoF with a total amplitude of 0.34 rad. Specifically, the viscoelastic characteristics of the wrist impedance in the flexion direction were calculated for angular perturbations started from the extended posture (+0.17 rad) to a flexed posture (−0.17 rad) and vice versa for those in the extension direction. The other two DoFs of the robot (RUD and PS) were held by the robot in the neutral position. The angular perturbations consisted of a minimum jerk profile, generated according to the following control law that was implemented by the microcontroller:


(1) $$\theta =\theta_{0}\pm △\,\left[6\,{\left(\frac{t}{T}\right)}^{5}-15\,{\left(\frac{t}{T}\right)}^{4}+10\,{\left(\frac{t}{T}\right)}^{3}\right]$$

where θ_0_ is the initial angular position (±0.17 rad), *t* is the time, Δ is the perturbation amplitude (0.34 rad) and *T* is the corresponding duration. Three values of *T* were used during the experiments: 200, 150, and 130 ms, with average velocities of 100, 130, and 150°/s, respectively.

Specifically, for each subject’s hand (RH, LH), the experimental protocol included nine different conditions given by the following combinations of perturbation velocities and grip demands:

- Three average velocity conditions: *slow* (100°/s), *medium* (130°/s), and *fast* (150°/s) perturbations.

- Three levels of grip: *self-selected grip* (requesting to hold a natural grasp), *medium grip* (approximately 40% of the MVC force grip force), and *high grip* (approximately 60% of the MVC force grip force).

These grip conditions were chosen to represent a range of grip capabilities and were confirmed to elicit differences in forearm muscle activity, as measured by sEMG. Throughout each condition of the experiment, subjects were asked to maintain the required grip force. In particular, during medium and high grip conditions, subjects received visual feedback of the total grip measured by the grip sensor through a virtual bar, displayed on the screen in front of them. The color of the bar changed if the total grip exceeded the target value. The color of the bar changed from yellow to blue if the total grip was maintained within a ±2% interval of the prescribed grip level. It is worth mentioning that the total grip measured by the sensor is a sum of the active grasping exerted by the subjects and the action of the *VELCRO*^®^ straps, carefully and firmly adapted to each subject in order to assure an approximately invariant sensor readout when subjects were requested to maintain a posture with the self-selected grip.

The protocol specified that, for each condition, there was a sequence of 56 trials (7 sets of 8 perturbations each), which lasted about 135 s, with a randomized delay between perturbations of approximately 2.3 ± 0.5 s (mean ± 1 SD). A rest time of 3 min was inserted between a condition and the next one, in order to prevent muscle and attentional fatigue. In particular, muscle fatigue was monitored by post-processing the sEMG signals recorded during the experimental conditions, as explained below.

The nine experimental conditions (for each hand) were randomly distributed on two different days (six random conditions on the first day and the remaining three on the second day) to reduce both central and/or peripheral fatigue. For every three conditions performed on the first randomly chosen hand, the setup was changed for the other hand and three random conditions were performed on that hand; then, the setup changed again (after almost 30 min) for the remaining conditions. However, to further reduce central and/or peripheral fatigue, the random selection algorithm of experimental conditions avoided to select two consecutive conditions with the same grip condition: in particular, we ensured that, for the same hand, there were not two consecutive conditions at the high grip level.

### Data Analysis

The torque transmitted by the robot to the hand for the FE perturbations was estimated directly by measuring the electrical current delivered by the control unit to the actuators and then applying a correction factor. Specifically, this correction factor accounted for belt-drive transmission and motor efficiency and was identified using a force-torque sensor. The current was sampled and recorded at a sampling rate of 1 kHz and smoothed by using a 1st order Savitzky–Golay filter, with a window of 45 samples (cut-off frequency: ∼20 Hz).

The control unit of the FE DoF was activated according to the position control mode, and was carefully tuned to achieve stable and accurate reproduction of the perturbation profile of Equation 1. In particular, accuracy was evaluated in several tests where we compared experimental results of the perturbation trajectories with the theoretical ones. We also evaluated how accurate the theoretical predictions of the velocity ($\dot{\theta }$) and acceleration ($\ddot{\theta }$) profiles were, expressed in Equation 2:


(2) $$\left\{ \begin{array}{c} \dot{\theta }=\pm △\,\left[30\,{\left(\frac{t}{T}\right)}^{4}-60\,{\left(\frac{t}{T}\right)}^{3}+30\,{\left(\frac{t}{T}\right)}^{2}\right]\\ \ddot{\theta }=\pm △\,\left[120\,{\left(\frac{t}{T}\right)}^{3}-180\,{\left(\frac{t}{T}\right)}^{2}+60\,\left(\frac{t}{T}\right)\right] \end{array}\right.$$

Because the robot did not provide a direct measurement of the end-effector velocity and acceleration, $\dot{\theta }$ and $\ddot{\theta }$ signals were differentiated by the recorded angular displacements and smoothed by using a 3rd order Savitzky-Golay filter, with a 45 ms windows (cut-off frequency: ∼12 Hz). Both torque and kinematic signals were re-sampled at the sEMG sample rate of 2048 Hz by linear interpolation.

### Wrist Mechanical Impedance Analysis

The purpose of this study was to evaluate the mechanical impedance of the wrist for the FE DoF by means of small amplitude, quick perturbations. The amplitude of the perturbations suggested to adopt a linear, second-order mass-spring-damper model that includes the moment of inertia of the wrist (*I*) and the corresponding viscoelastic properties of the neuromuscular apparatus of the wrist: damping (*B*) and stiffness (*K*) parameters (Kearney and Hunter, 1982; Tsuji et al., 1995). However, the perturbation method adopted in this study can only provide an estimate of the total mechanical impedance of the system, that includes the wrist and the robot. By adopting the same mass-spring-damper model for both components, the equivalent total mechanical impedance equation can be written as:


(3) $$\tau -\tau_{0}=I_{e\,q}\,\left(\ddot{\theta }-{\ddot{\theta }}_{0}\right)+B_{e\,q}\,\left(\dot{\theta }-{\dot{\theta }}_{0}\right)+K_{e\,q}\,\left(\theta -\theta_{0}\right)$$

where


$$\left\{ \begin{array}{c} I_{e\,q}=I_{r\,o\,b\,o\,t}+I_{w\,r\,i\,s\,t}\\ B_{e\,q}=B_{r\,o\,b\,o\,t}+B_{w\,r\,i\,s\,t}\\ K_{e\,q}=K_{r\,o\,b\,o\,t}+K_{w\,r\,i\,s\,t} \end{array}\right.$$

τ (Nm) is the time-varying torque exerted by the robot motors to the FE DoF during the perturbation, derived from the current delivered by the control unit to the motors, and τ_0_ is the torque value maintained before initiating the perturbation; θ (rad) is the angular displacement of the perturbation, computed in agreement with Equation 1 and measured by the encoder of the FE motor; $\dot{\theta }$ is the corresponding rotational velocity (rad/s) and $\ddot{\theta }$ is the rotational acceleration (rad/s^2^), estimated according to Equation 2; θ_0_ is the initial angular value, which is equal to 0.17 rad, if the perturbation proceeds from the extension to flexion, and −0.17 rad in the opposite case; ${\dot{\theta }}_{0}$ and ${\ddot{\theta }}_{0}$ are the initial values of the angular velocity and acceleration, respectively, and have both null values in the adopted experimental protocol.

The mechanical impedance modeled by Equation 3 is a linear combination of the intrinsic mechanical impedance of the device and the corresponding impedance of the wrist. A substantial part of the recorded torque resulted from the dynamics of the device and an estimation of this behavior was made before the experiments (see below for more details). Such “device torque” was subtracted from the recorded torque to get the “wrist torque,” which was then subjected to further analysis.

More specifically, the “best fit” parameters of the total mechanical impedance (*I*_*eq*_, *B*_*eq*_, and *K*_*eq*_) were estimated by using a least square approximation (LSA) procedure, capable of minimizing the mean-square error between the torque recorded experimentally and that predicted by the model. In order to discriminate the contribution of the device from the biological component, we performed the LSA in two experimental contexts: (1) *robot alone*, having disconnected the hand from the device and (2) *robot + hand*, both during the experimental conditions characterized by the three different velocity perturbations. In the former context, the LSA procedure was repeated for the three velocity conditions defined above (slow, medium, and fast). In the latter context, the procedure was applied to the nine conditions of the experimental protocol.

For each experimental condition, we excluded from the analysis the perturbations whose torque signals differed significantly (zscore > 2.5) from the others of the same type of movement (flexion and extension). Then, we calculated *B* and *K* for each movement and within-subject we averaged the values to obtain a single value of *B* and *K* for each type of movement (flexion and extension). The same procedure of averaging was used across-subjects, thus producing final values of the *B* and *K* parameters for each type of movement in each experimental condition.

### Surface Electromyography and Grip Analysis

The raw sEMG signals were band-pass filtered (10–400 Hz) with a 4th order Butterworth filter, full-wave rectified and low-pass filtered with a 4th order Butterworth filter with a 9 Hz cut-off frequency to obtain linear envelopes (Chalard et al., 2020). For each subject and each hand, sEMG envelopes were normalized to their corresponding maximum value obtained during the MVE and expressed as %MVE. In particular, the maximum baseline value corresponds to the peak from the filtered MVC data.

The duration of the experimental sessions was short, and a rest time was provided among conditions to mitigate muscle fatigue. However, since muscular fatigue is known to affect biomechanical properties, we integrated a tool for monitoring the onset of fatigue by investigating the trend of the mean frequency (MF) of the sEMG spectrum of each muscle (Cifrek et al., 2009) during the experimental protocol. Each experimental condition was divided into five phases, approximately 25 s each, and a single value of MF was computed in each phase. The MF extracted during the first phase of the first random experimental condition was considered the baseline MF. For each participant, MF data were normalized with respect to the corresponding baseline MF and, as fatigue occurred, the power of the sEMG signals tended to shift toward lower frequencies (Merletti et al., 1990). In agreement with (Mugnosso et al., 2019), the onset of fatigue was detected by a decrease in MF below 25% of the corresponding baseline MF.

For all subjects, we evaluated the average grip magnitude during three different phases of each experimental condition: (1) 200 ms before the onset of the perturbation (*G*_0_); (2) during the perturbation (Δ*G*); (3) from the end of perturbation to 300 ms after the perturbation (*G*_*f*_).

### Statistical Analysis

First, we investigated that the three chosen levels of grasping were significantly different from each other by using a Wilcoxon rank sum test on the amount of sEMG muscle activity required to perform those conditions. After, we performed a global goodness-of-fit test (*R*^2^) of the resulting mathematical model of wrist mechanical impedance (Equation 3). Then, we performed the same statistical analysis on both the dynamic stiffness (*K*) and damping (*B*) parameters. First, we evaluated the distribution of *K* parameter through a Lilliefors test and then analyzed any statistical relationships in the data belonging to different experimental conditions using proper statistical tests. Specifically, a Wilcoxon rank sum test was used to check any statistical difference of *K* (and *B*) parameters computed both in extension and flexion movements, while a one-way analysis of variance (ANOVA) non-parametric test (Kruskal–Wallis) was used to test for differences in the experimental conditions. Finally, to test the statistical effect of the grip percentage on the *K* (and *B*) values, we performed a mixed-effects model with *K* (and *B*) as the dependent variable, grip percentage as the independent variable, and subject as the random factor. We further investigated the behavior of *K* (and *B*) between the two hands using a Wilcoxon rank sum test. Jamovi Statistical Data Analysis tool (JSDA, version 1.6.23) and MATLAB R2020b were used to conduct statistical analysis.

## Results

### Preliminary Tests and Analysis

The first preliminary analysis was focused on the evaluation of the intrinsic robot parameters by means of the *robot-alone* perturbation, described in the methods. The following results were obtained: *I*_*robot*_=0.017kgm^2^, *B*_*robot*_=0.22Nms/rad, *K*_*robot*_=0.14Nm/rad. As a cross-check of the consistency of the method, we verified that the parameter estimates were invariant with respect to perturbation velocity (slow, medium, and fast) and direction (flexion and extension). Next, we evaluated the hand moment of inertia (*I*_*hand*_), considering that the inertia evaluated by the LSA method in the *robot+hand* condition is the sum of the two elements: *I*_*eq*_=*I*_*robot*_ + *I*_*hand*_. In order to compute the *I*_*hand*_ we considered a document containing anthropometric and biomechanical parameters of the human body (Anthropology Research Project, 2000) including the moment of inertia, the center of mass and the mass of the hand. In particular, using the Huygens-Steiner theorem, we calculated the *I*_*hand*_ parameter about the FE axis of the hand obtaining the following average value: *I*_*hand*_=0.0019kgm^2^. Thus, hand inertia contributed no more than 10% to the overall inertia. We also evaluated the level of static friction that might affect the robot, by performing *robot-alone* perturbations at a very slow, constant speed (1°/s). The RMS of the residual torque was rather low (0.2 Nm), as expected considering the careful mechanical re-design of the WristBot used in the study.

We further compared the torque required to overcome wrist joint inertia with the torque required to overcome wrist joint stiffness in the tested population during the self-selected grip and slow velocity experimental condition. The computed average value of the inertial component of the wrist was 0.07 Nm, the viscous component was 0.05 Nm, and the elastic component was 0.15 Nm.

**FIGURE 2:**
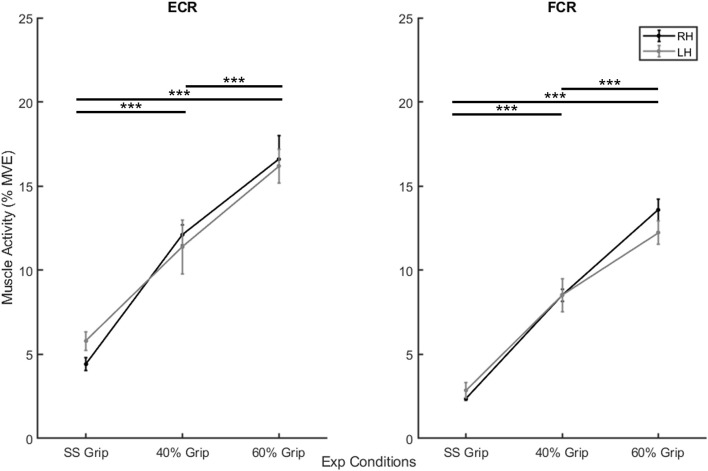
Mean muscle activity (%MVE) for extensor carpi radialis (ECR) (left) and flexor carpi radiali (FCR) (right). Black and gray lines represent RH and LH group, respectively. Mean ± std. ***, statistically significant, *p* < 0.001.

In the second preliminary check, we analyzed the level of grip required and the amount of sEMG activity used during the selected experimental conditions. In particular, Table 1 shows the average grip force (%MVC) maintained during the 200 ms interval before the delivery of the perturbation in the three grip conditions (self-selected grip: SS Grip, 40% MVC Grip, and 60% MVC Grip), for both hands (RH, LH). Moreover, Figure 2 displays the corresponding normalized muscle activity of FCR and ECR for the same grip conditions. The Wilcoxon rank sum test assessed that, in both hands, FCR and ECR muscle activity differed significantly between SS Grip and 40% Grip (FCR: *W* = 4.0 *p* < 0.001 in RH, *W* = 25.0 *p* < 0.001 in LH; ECR: *W* = 42.0 *p* < 0.001 in RH, *W* = 70.0 *p* < 0.001 in LH), between SS Grip and 60% Grip (FCR: *W* < 1.0 *p* < 0.001 in RH, *W* = 6.0 *p* < 0.001 in LH; ECR: *W* = 12.0 *p* < 0.001 in RH, *W* = 15.0 *p* < 0.001 in LH), and finally between 40 and 60% Grip (FCR: *W* = 9.0 *p* < 0.001 in RH, *W* = 13.0 *p* < 0.001 in LH; ECR: *W* = 16.0 *p* < 0.001 in RH, *W* = 17.0 *p* < 0.001 in LH).

**TABLE 1:** Average grip sensor level [% maximum voluntary contraction (MVC) Grip Force] during the 200 ms before the delivery of the perturbation in the three grip conditions (SS Grip, 40% and 60% of MVC Grip Force), for both hands (right hand: RH, left hand: LH).

	**SS Grip**	**40% MVC Grip**	**60% MVC Grip**
RH	4.7% ± 2.8%	40.3% ± 0.5%	60.3% ± 0.5%
LH	3.7% ± 2.2%	40.4% ± 0.4%	60.1% ± 0.5%

After the preliminary evaluations, we focused on the estimation of the viscoelastic properties of the mechanical impedance of the wrist, for the different velocity perturbations and grasping conditions. To consolidate the robustness of this assessment method, three evaluations were required to characterize the robustness of the method: (1) evaluating the reliability of the mathematical model, expressed by Equation 3; (2) assessing the ecological-ergonomic characterization of the experimental protocol, described in the previous section; (3) estimating the stability of the grasping paradigms.

**FIGURE 3:**
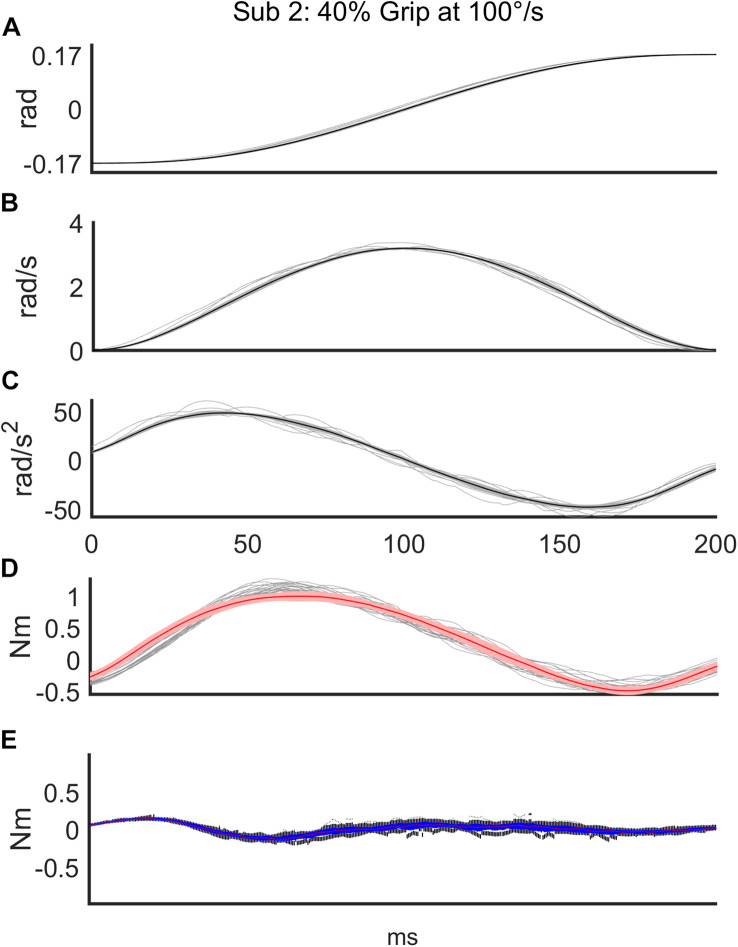
Representative subject’s (Subject 2) recorded signals for a sequence of 56 trials in the condition of slow (100°/s) perturbations at 40% Grip. The top three subplots show the time course of angular displacement **(A)**, velocity profile **(B)**, and acceleration profile **(C)** of the perturbations (thin: measured values; thick: mean value). The bottom two subplots demonstrate the power of the curve fitting of the model-estimated torque profile (red line) on top of the measured torque profiles (gray lines) **(D)** and the time-course of the discrepancy between the two torque profiles **(E)**.

Regarding the first analysis, we evaluated the discrepancy between the torque signals generated by the robot and the torques reconstructed according to the mathematical model of Equation 3. The top three subplots of Figure 3 show a representative example of one subject’s kinematics during the slow perturbations (100°/s) and 40% Grip. The bottom two subplots of Figure 3 demonstrate the power of the curve fitting approach for the model-estimated torque profile. *B* and *K* values were calculated by the LSA procedure, over the recorded torque profiles. More generally, the goodness-of-fit (*R*^2^) between the model-generated and experimental torque profiles, evaluated for the subjects performing the nine different experimental conditions, is reported in Table 2, limited to the subjects belonging to the RH group. The *R*^2^ obtained as the result of fitting the mathematical model to the real data under different experimental conditions were higher than 0.88, showing how well this chosen model fits the outline of our data. Specifically, we averaged the *R*^2^ across subjects and we obtained a high correlation for the condition combining fast perturbations with 60% grip condition (*R^2^* = 0.88)while the greatest correlation was found for the condition of slow perturbations at self-selected grip (*R*^2^ = 0.98). The same analysis was also carried out for the subjects of the LH group and as expected, no significant difference was found.

**TABLE 2:** Goodness-of-fit (*R*^2^) between the model-generated and experimental torque profiles, evaluated for all subjects belonging to the RH group and performing the nine experimental conditions divided by the direction of the perturbation.

			**Speed conditions**		
			**Fast**	**Medium**	**Slow**
Grip conditions	SS Grip	Extension	0.91 ± 0.04	0.94 ± 0.05	0.98 ± 0.02
		Flexion	0.91 ± 0.03	0.94 ± 0.05	0.98 ± 0.02
	40%	Extension	0.90 ± 0.06	0.93 ± 0.05	0.97 ± 0.03
		Flexion	0.88 ± 0.06	0.93 ± 0.04	0.96 ± 0.04
	60%	Extension	0.88 ± 0.06	0.92 ± 0.05	0.97 ± 0.03
		Flexion	0.88 ± 0.06	0.92 ± 0.07	0.96 ± 0.04

**FIGURE 4:**
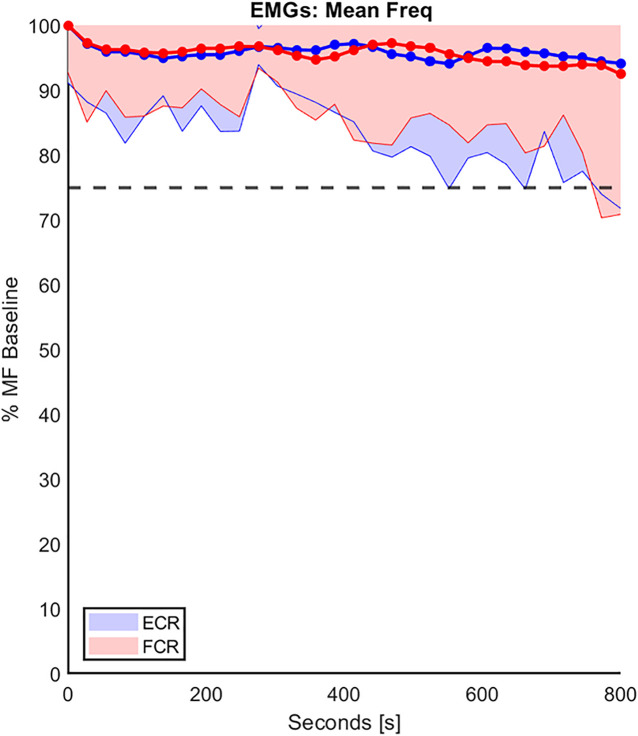
The trend of the percentage decline of the mean frequency (MF) of both FCR and ECR (red and blue, respectively), over the duration of the experiment protocol in the RH group. The MF values were normalizated to the corresponding baseline MF performed on the first day of experimentation. These data were averaged on the entire population and the standard deviation (shaded part) is provide. The horizontal line represents the 25% threshold used in this work to be considered a fatigue cut-off.

Regarding the second analysis, namely the ecological-ergonomic characterization of the experimental protocol, we wished to assure that the protocol was well accepted by the subjects (a cognitive requirement) and did not produce muscle fatigue (a biomechanical requirement). The former requirement was tested by asking the subjects, before and after the protocol: all of them confirmed that they had a clear understanding of the rationale of the experiment and felt at ease with the experimental setup. For the latter requirement, we focused on the possible spectral shift of the sEMG signals of MF as explained in the methods. Figure 4 shows an example of the trend of the percentage of MF of both FCR and ECR, over the duration of the experimental protocol, compared with the corresponding baseline MF performed on the first day of experimentation, in the RH group and averaged among subjects. In the figure, the standard deviation of the data is shown (shaded part) to provide an overview of the variability of the group. At the end of the experiment, the mean decrease in the MF of both muscles for both RH and LH groups was at most around 10% of their baseline value, confirming that physical effects of fatigue were negligible.

**FIGURE 5:**
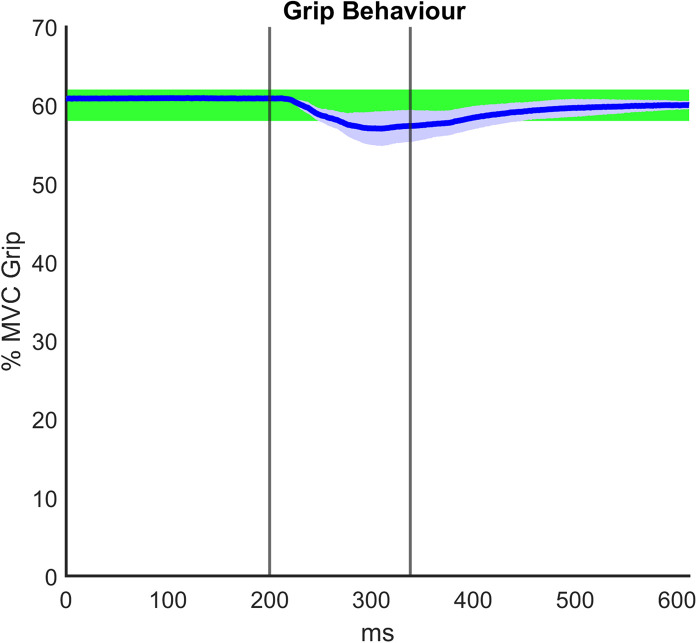
Representative grip force data averaged among subjects and over time during the fast perturbation at 60% Grip, spanning from 200 ms pre perturbation to 300 ms after the end of the perturbation (130 ms). The graph displays the visual bar at 60% maximum voluntary contraction (MVC) Grip Force, as it was shown to the participants: the percentage of the required grip with its tolerance range (±2%) is confined to the green area. The vertical lines identify the duration of the fast perturbations (specifically, 130 ms).

Regarding the grip paradigm test, we verified to what extent the grip remained constant during the perturbation as a consequence of the good mechanical coupling between hand and handle. Moreover, in post-processing, we verified that the muscle activity before the onset of each perturbation also did not significantly differ. Figure 5 illustrates a representative temporal evolution of the grip sensor data during the fast perturbation at 60% Grip, spanning from 200 ms pre perturbation to 300 ms after the end of the perturbation (130 ms). In particular, during the perturbation there was a small decrease in the grip (around 4%), which was approximately recovered after a few hundred milliseconds. This decrease in grip could be due to the soft sensor itself and the elasticity of the skin. Grip magnitude was averaged among subjects, computed in the three different phases of perturbations (*G*_0_, Δ*G*, *G*_*f*_) and shown in Table 3. Table 3 confirms a consistent grip force in all the experimental conditions for both RH and LH groups. This preliminary assessment is quite relevant, as clarified in the following analysis of the calculation of the viscoelastic parameters.

**TABLE 3:** Average grip force (%MVC) computed in the three different phases of perturbations (*G*_0_: during 200 ms before the onset of the perturbation; Δ*G*: during the perturbation; *G*_*f*_: from the end of perturbation to 300 ms after the perturbation) in the 40 and 60% grip conditions.

	**G_0_**		△**G**		**G_f_**	
	**RH**	**LH**	**RH**	**LH**	**RH**	**LH**
40% Grip	41.11% ± 0.02%	41.26% ± 0.02%	36.09% ± 1.55%	35.04% ± 1.99%	39.81% ± 0.17%	39.78% ± 0.08%
60% Grip	60.9% ± 0.02%	60.85% ± 0.03%	57.62% ± 0.95%	56.82% ± 1.26%	59.94% ± 0.14%	59.84% ± 0.28%

### Main Results: Viscoelastic Parameters of the Wrist Mechanical Impedance

Once we verified that the experimental protocol did not induce measurable levels of fatigue and the subjects succeeded to keep an approximately consistent grip level during the experimental conditions, we could proceed with the analysis of the results, focused on the calculation of the viscoelastic parameters: such parameters of the wrist mechanical impedance, evaluated by the LSA method for each experimental condition, are reported in the Tables 4, 5.

**TABLE 4:** Stiffness parameter K (Nm/rad) for the RH of the healthy population and for the nine experimental conditions.

			**Speed conditions**		
			**Fast**	**Medium**	**Slow**
Grip conditions	SS Grip	Extension	0.49 ± 0.32	0.43 ± 0.24	0.59 ± 0.18
		Flexion	0.48 ± 0.27	0.52 ± 0.20	0.61 ± 0.26
	40%	Extension	1.48 ± 0.49	1.30 ± 0.57	1.33 ± 0.36
		Flexion	1.41 ± 0.47	1.45 ± 0.52	1.33 ± 0.37
	60%	Extension	1.84 ± 0.39	1.71 ± 0.56	1.68 ± 0.45
		Flexion	1.80 ± 0.35	1.89 ± 0.46	1.69 ± 0.46

**TABLE 5:** Damping parameter B (Nms/rad) for the RH of the healthy population and for the nine experimental conditions.

			**Speed conditions**		
			**Fast**	**Medium**	**Slow**
Grip conditions	SS Grip	Extension	0.05 ± 0.02	0.03 ± 0.02	0.02 ± 0.01
		Flexion	0.06 ± 0.02	0.05 ± 0.02	0.03 ± 0.02
	40%	Extension	0.04 ± 0.02	0.03 ± 0.01	0.03 ± 0.02
		Flexion	0.04 ± 0.02	0.04 ± 0.01	0.03 ± 0.02
	60%	Extension	0.04 ± 0.02	0.03 ± 0.02	0.03 ± 0.02
		Flexion	0.04 ± 0.01	0.04 ± 0.02	0.03 ± 0.02

The wrist damping parameter (*B*) had values in the range of 0.02–0.06 Nms/rad. These values were consistent under the different experimental conditions, i.e., the values did not differ significantly under the different velocity and grip conditions.

**FIGURE 6:**
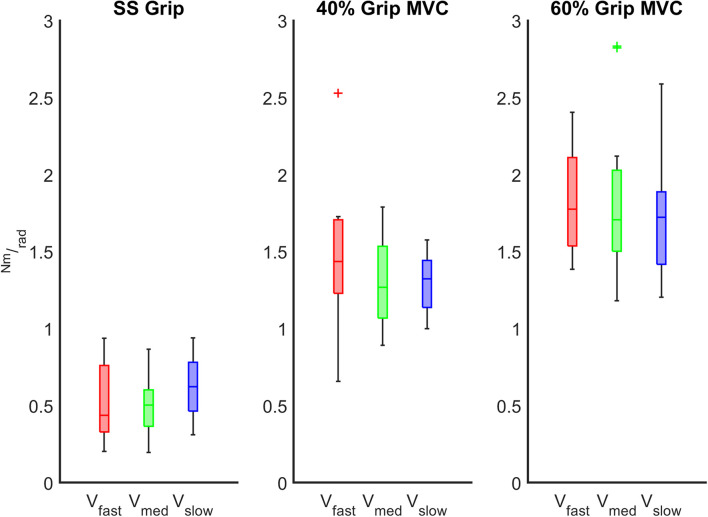
*K* (Nm/rad) calculated across the different velocity conditions at the fixed grip conditions (SS Grip, 40% and 60% MVC Grip Force represented in the left, center and right subplots, respectively). These data belong to the RH group. The red, green, and blue boxplots identify the experimental conditions at fast, medium, and slow perturbations, respectively.

**FIGURE 7:**
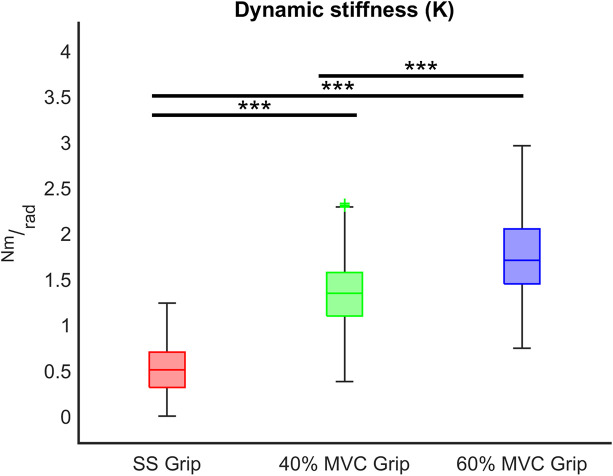
*K* (Nm/rad) across different grip conditions (SS Grip, 40% and 60% of MVC Grip Force). These data belong to the RH group. ***, statistically significant, *p* < 0.001.

The characterization of the wrist stiffness parameter (*K*) is reported in Figures 6, 7. First, we tested the non-normality distribution of the data through a Lilliefors test. For each hand, a Wilcoxon rank sum test assessed that globally (i.e., considering all the experimental conditions), there was no significant difference in *K* values between extension and flexion movements and averaged among subjects (RH: *p* = 0.72, LH: *p* = 0.80). We further verified that the nonsignificant difference persisted within each experimental condition. Because of this result, for the following analysis, in each subject we considered the *K* values calculated for each movement independently of the direction of the perturbation. Second, we analyzed the significant difference of the *K* values across the velocity perturbations at the same grip condition. The Kruskal–Wallis test reported the same statistical results for both hands: no significant differences in *K* values were found across the different velocity perturbations in either the SS Grip (RH: χ^2^ = 2.63 *p* = 0.27, LH: χ^2^ = 1.01 *p* = 0.60), the 40% Grip (RH: χ^2^ = 0.93 *p* = 0.63, LH: χ^2^ = 0.44 *p* = 0.80), and the 60% Grip (RH: χ^2^ = 0.49 *p* = 0.78, LH: χ^2^ = 0.65 *p* = 0.72) conditions (Figure 6).

The extent to which the results summarized above on wrist stiffness for FE movements were influenced by the rapid motor responses (traditionally called reflexes) may be questioned. After the onset of the perturbation movement, these rapid motor responses are in the range of 20–105 ms in the stretched muscle (Kuczynski et al., 2017). To answer this question, we performed only a qualitative observation of the sEMG signals of the stretched muscles and they did not show any systematic stretch reflex. This might lead us to consider the estimated wrist stiffness as a consequence of the mechanical properties of the stretched muscles, for the three different levels of tonic muscle activity, detected by the grip sensor. However, this consideration needs further analysis to be concluded; for now, we can finalize that the estimated stiffness simply reflects a combination of intrinsic properties and reflex activity.

**FIGURE 8:**
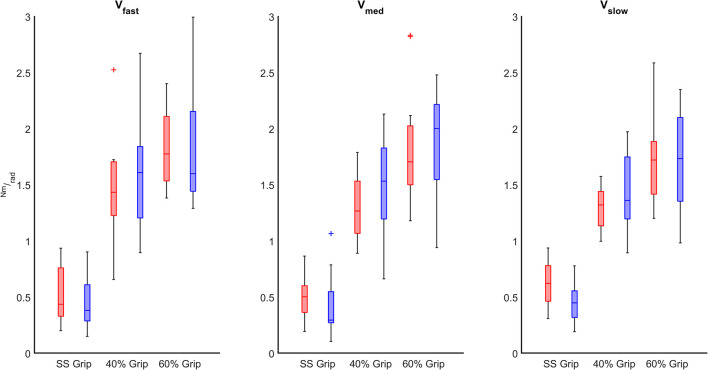
*K* (Nm/rad) computed in both RH (red) and LH (blue) groups across different grip conditions considering the velocity of the perturbations (fast, medium, slow represented in the left, center, and right subplots, respectively).

Finally, we evaluated the influence of the grip intensity on the *K* parameter. Based on the previous results, in each grip condition, we considered the *K* values calculated for each subject movement, independently of the direction of the perturbations and the velocity. In particular, we calculated the relationship between the grip conditions and the *K* values through a mixed model. Results reveal a significant positive relationship between grip demands and *K* values for both hands (*B* = 0.02 *p* < 0.001). A *post-hoc* Bonferroni test revealed that the significant difference between grip demands and *K* values reflected a statistically significant difference in grip conditions: between 40% Grip and SS Grip (RH: *B* = 0.87 *p* < 0.001, LH: *B* = 1.16 *p* < 0.001), between 60% and SS Grip (RH: *B* = 1.27 *p* < 0.001, LH: *B* = 1.47 *p* < 0.001) and between 40 and 60% Grip (RH: *B* = 0.41 *p* < 0.001, LH: *B* = 0. 31 *p* < 0.001) conditions (Figure 7). Interestingly, these results were the same for both hands. Indeed, a Wilcoxon rank sum test assessed that globally there was no statistically significant difference (χ^2^ = 0.05 *p* = 0.83) between the *K* values computed in the right and left hands (Figure 8).

## Discussion

The purpose of this work was twofold: (1) to build and validate a mathematical model that could evaluate the viscoelastic properties of both wrists in a healthy, right-handed population, using a robotic device designed for the biomechanical analysis and motor rehabilitation of the wrist; (2) to explore the relationship between viscoelastic parameters of the wrist joint, perturbation velocity and grip force. To meet both objectives, we designed an experimental protocol consisting of sequences of small amplitude and fast angular perturbations of different velocities along the flexion-extension DoF of the wrist, combined with different grip force requirements exerted on the robotic handle. The selection of specific perturbation parameters (displacement range and velocity) allowed us to model the relationship between the torques exerted by the robot and the kinematics of the wrist as a linear, second-order mass-spring-damper system. Starting from the mathematical model, we extracted the viscoelastic properties of the wrist joint using a least-square approximation method. The model fits well with the recorded torque profiles of the robotic device. In agreement with the findings of Charles and Hogan (2011) we found that the rotational dynamics of the wrist are dominated by joint stiffness, not wrist inertia, at least for the type of quick movements investigated in our study.

In this framework, special attention was addressed to the proper acceptance of the different experimental conditions by the subjects, with the prerequisites of a firm connection between the hand and the robotic handle, consistent and low variability in grip among conditions and negligible neuromuscular fatigue. To properly evaluate the viscoelastic properties of the wrist, as an important first step, we wanted to eliminate the potential confounding effects of muscle fatigue on wrist parameters. Thus, we let the participant rest after each condition, we changed the setup for the other hand after three conditions on the current hand, and we did not have consecutive conditions with the same percentage of the grip. It may be possible that muscles primarily involved in the grasping action could have experienced fatigue in this protocol. The most affected muscles during gripping may be those serving the fingers; in fact, Hazelton et al. (1975) showed that the fourth finger, served by the flexor digitorum superficialis (FDS), contributes 25–28% of the total gripping force. In our work, the recorded muscles (FCR, ECR) predominantly generate wrist actions and we are confident (Figure 4) that fatigue of these muscles were minimized during the protocol.

The dynamic stiffness computed in both extension and flexion movements was not statistically different. This represents an interesting result, already presented in Feldman and Levin (1995); Pando et al. (2014), but different with what was found by Formica et al. (2012), who evaluated that the stiffness in extension was statistically greater than stiffness in flexion. Explanations for this result, and for the discrepancy in the literature, could be traced to the choice of the natural position to calculate the viscoelastic properties of the wrist in both flexion and extension directions. In fact, as explained in the methods, in our study, the subject received angular perturbations that moved the wrist from an extended to a flexed position and vice versa. Although the chosen starting angle was very small (0.17 rad), a comparison with these studies, in which the estimated viscoelastic properties of the wrist in the flexion and extension directions were computed from the natural position to the chosen angular posture and back to the start, may be not accurate. Additionally, the selected velocity conditions of the current study were higher than the one used for the perturbations in Formica’s study (60°/s). However, to allow a comparison between the stiffness values estimated in the two studies [this work (TW) and Formica’s (FW)], we consider the estimates computed in the slowest velocity condition (100°/s) at the self-selected grip: toward flexion 0.53 ± 0.38 (TW) vs. 0.55 ± 0.17 (FW); toward extension 0.45 ± 0.17 (TW) vs. 1.02 ± 38 (FW).

Notably, the second main finding of our study was that the dynamic stiffness did not significantly change across different perturbation velocity conditions. Previously, De Vlugt et al. (2011) studied the effects of wrist joint rotation velocity on the short-range stiffness (SRS) and on the elastic limit, which represents the point where the stiffness drops and the muscle begins to behave as a viscous damper. The authors reported that the elastic limit increased significantly with joint velocity, whereas the SRS did not change with angular velocity. To date, there is no other work on wrist joint stiffness studied under different velocity conditions that could support our finding. In another study, Lee et al. (2002) investigated the impact of stretch velocity on the reactive torque for the elbow joint in a healthy population. They showed an increased reactive torque associated with an increased stretch velocity in hemiparetic spasticity and parkinsonians rigidity, but not in normal muscle tone. Interestingly, given no significant differences for stiffness calculated in extension and flexion directions and across velocity conditions, we considered stiffness values independent of both these parameters for healthy subjects.

A significant positive relationship between stiffness parameters and grip intensity for both hands was found. In particular, we found an increasing trend of stiffness as the grip force demand increased. Similarly, Holmes et al. (2015) found that increased grip force led to an increase in forearm muscle co-contraction, which led to an increase in individual muscle contributions to total joint rotational stiffness around the FE direction of the wrist joint prior to unexpected perturbations. However, the increase in wrist stiffness with muscle activity is well known in the literature and has been linked to an increase in the number of cross bridges (Hunter and Kearney, 1982; De Vlugt et al., 2011). Therefore, in connection with this work, we have demonstrated that increases in grip force largely influences wrist joint stiffness, suggesting the importance of incorporating an accurate monitoring of the grip force during wrist stiffness measurement protocols. In previous works on the evaluation of the viscoelastic properties of the wrist joint, the measurement of the grip force was never directly evaluated. Of course, other variables that could be related to grip force, such as the muscle activation, have been considered. Therefore, one of the novelties of this work was in controlling for grip force instead of muscle activity.

What clearly emerged was that the stiffness values computed in the dominant and nondominant hands did not differ significantly in all the experimental conditions in which the healthy population was tested. The only difference between the two measurements was that the stiffness values computed for the LH were characterized by larger variability than those computed for the right hand. Greater wrist control of the dominant hand could account for this difference (Heuer, 2007). However, the absence of significant difference of stiffness parameters between hands, already demonstrated in the right-handed male population by Durand et al. (2019), represents an interesting finding that could be projected into the clinical context in studies involving neuromuscular impairments. Specifically, using our experimental protocol, researchers could quickly record data from both patient hands (e.g., affected and unaffected) in a controlled and consistent approach to objectively investigate clinical outcomes.

Turning to the damping parameter, the experimental results showed low variability among subjects with values that were consistent across the entire set of experimental conditions in both FE directions. In the literature, very few studies systematically measured the damping parameter for the wrist joint. The damping values found in our study are in line with those reported in the literature (Gielen and Houk, 1984; Klomp et al., 2014). In our study, no dependence of the damping parameters with changes in the velocity perturbation was shown, but as explained in the section “Limitations and Future Works,” the range of mean velocity conditions chosen was relatively small. However, as evidenced by previous research on the human ankle dynamics, both damping and stiffness parameters of the joint are strongly related to the mean torque level (Hunter and Kearney, 1982; Weiss et al., 1988); in particular, the stiffness parameter varies linearly with the mean torque whereas the damping varies more slowly, as the square root of the mean torque. In this study, we showed that as the grip condition increased, the wrist stiffness coefficient increased, whereas the wrist damping coefficient did not change significantly. This latter result had already been presented for the wrist joint in Milner and Cloutier (1998) during voluntary movements and in Klomp et al. (2014) during displacement perturbations in the FE direction. In our case, possible explanations for this result can be attributed to a small difference in the mean torque recorded at the wrist joint under the different grip conditions, and not distinguishing the contribution between intrinsic and reflex to the damping coefficient. However, this represents the relevant physiological baseline of healthy subjects, against which to evaluate the responses of subjects with exaggerated reflex activation of muscles, after controlled mechanical disturbances.

### Limitations and Future Works

The main limitation of our study is that the evaluation of the viscoelastic properties of the wrist was constrained to a single DoF (FE direction), preventing discussion with other interesting works. Specifically, several papers computed the viscoelastic properties at the wrist joint along different directions. They all concluded that the minor axis of the stiffness was directed along radial extension–ulnar flexion, a direction known as the motion of dart thrower’s wrist and oriented obliquely to the anatomical axes (Crisco et al., 2011; Formica et al., 2012; Erwin et al., 2021). Thus, it would be interesting to test our protocol along the other directions of wrist movement and try to compare the results. In fact, our next step will be to implement the experimental protocol with the robotic device for 2D or 3D planes, involving the RUD and/or PS DoF, by extending the experience gained with the same device for 1D-2D-3D evaluations of wrist proprioception (Cappello et al., 2015; Marini et al., 2018; Albanese et al., 2021). However, even the choice of average velocity conditions from 100 to 150°/s is a relatively small range. This could be the cause of not observing a difference on the viscoelastic parameters across the velocity conditions. Thus, we should consider repeating the same protocol using significantly slower or faster perturbations. Furthermore, our study focused on analyzing the stiffness parameter as a combination of intrinsic properties and reflex activity (if any) and not a distinction between the two. Thus, our next step could be to analyze the sEMG signals of the muscles involved in the experimental protocol in terms of short-, long-latency stretch responses and link the reflex response to the intrinsic variables.

## Conclusion

In this work, our main goals were to build a mathematical model to extract viscoelastic characteristics of both wrists in a healthy population and relate them to different perturbation velocities and grip demands. First, we demonstrated that the damping parameter did not change under the chosen experimental conditions; whereas the stiffness parameter was independent of the direction and velocity of the perturbation, but it increased as the grip demand increased. Finally, both damping and stiffness parameters did not differ significantly between both hands (dominant and nondominant).

The novelty of our research lies in the ability to accurately quantify the amount of grip force exerted by the subject on the robotic handle wrapped by a soft grip sensor. With this study, we demonstrated that increases in grip force largely influenced wrist joint stiffness, concluding that grip force feedback should be incorporated into the stiffness measurement protocol by making it a more controlled variable. The actual measurement of the grip force becomes very useful also in light of extending the presented experimental protocol to clinical settings. Indeed, in this study, we altered grip to effectively stiffen the joint and we were able to evaluate the corresponding muscle responses and effects grip force had on wrist joint stiffness. Moreover, patients with abnormal muscle tone may have a different level of grasping ability than the healthy population. For this reason, having a tool that allows the monitoring of this characteristic is very useful in understanding the viscoelastic properties of the muscles involved and if and how they change over an assessment.

## Data Availability Statement

The raw data supporting the conclusions of this article will be made available by the authors, without undue reservation.

## Ethics Statement

The studies involving human participants were reviewed and approved by the Local Ethical Committee of Liguria Region (n.222REG2015), in accordance with the Declaration of Helsinki. The patients/participants provided their written informed consent to participate in this study. Written informed consent was obtained from the individual(s) for the publication of any potentially identifiable images or data included in this article.

## Author Contributions

VF, PM, JZ, and MH formulated the experimental question and design the study. VF collected the data, performed the data analysis and statistics, and wrote the manuscript. VF, MH, LM, PM, and JZ participated in the results interpretation. JZ supervised the study and revised the final version of the manuscript. All authors contributed to the article and approved the submitted version.

## Conflict of Interest

The authors declare that the research was conducted in the absence of any commercial or financial relationships that could be construed as a potential conflict of interest.

## Publisher’s Note

All claims expressed in this article are solely those of the authors and do not necessarily represent those of their affiliated organizations, or those of the publisher, the editors and the reviewers. Any product that may be evaluated in this article, or claim that may be made by its manufacturer, is not guaranteed or endorsed by the publisher.
